# Treatment of Acute Myeloid Leukemia in the Elderly

**DOI:** 10.3390/hematolrep18050070

**Published:** 2026-09-28

**Authors:** Harsh Vadnagara, Amol Dua, Sahil Garg, Pranay Juneja, Abhay Singh, Akriti G. Jain

**Affiliations:** 1 B.J. Medical College & Civil Hospital, Ahmedabad 380016, India; 2 Anne Arundel Medical Center, Annapolis, MD 21401, USA; amol117dua@gmail.com (A.D.);; 3 Postgraduate Institute of Medical Education and Research, Chandigarh 160012, India; pranayjuneja2000@gmail.com; 4 Taussig Cancer Institute, Cleveland Clinic, FACP 9500 Euclid Avenue, CA-60, Cleveland, OH 44195, USA

**Keywords:** AML, elderly, intensive chemotherapy (IC), hypomethylating agents, novel therapeutics

## Abstract

**Background:** Acute myeloid leukemia (AML) is the most prevalent type of leukemia, which primarily affects older patients. Because the definition of ‘elderly’ AML varies across the literature, from ≥60 years in some cooperative-group studies to a strict ≥ 75-year threshold in the pivotal VIALE-A trial, we adopt ≥ 60 years as our working definition. Despite recent advancements, the 5-year overall survival (OS) of AML patients is just 30% and declines to 5–10% in patients older than 65 years at diagnosis. The main objective of treatment in older individuals is to achieve complete remission (CR) and maintain a good quality of life. **Discussion:** Traditionally, intensive chemotherapy (IC) has been used for management of AML since the 1970s. Patients older in age are often not eligible for IC due to factors such as advanced age, comorbidities, and disease characteristics such as high-risk cytogenetics and adverse risk mutations. When considering patients for intensive (and alternative) chemotherapy and allogeneic hematopoietic stem cell transplantation (alloHSCT), we need to consider a standardized fitness assessment tool, which would significantly improve our capacity to individualize treatment strategies and design patient-specific clinical trials. Tools such as performance status, the Ferrara criteria, and comprehensive geriatric assessment (CGA) can help evaluate the baseline comorbidity burden in elderly patients. The discovery of hypomethylating agents (HMAs) like azacitidine and decitabine and BCL-2 inhibitors such as venetoclax has been pivotal in the treatment of elderly AML patients unfit for IC. As our knowledge advances in understanding the pathogenesis of AML, we are learning about new genetic mutations and molecular targets that play an important role in the development of AML. In this revised review, we explicitly separate drugs that are FDA-approved and have been studied specifically in older or unfit AML patients from drugs that are approved but lack dedicated efficacy data in this population and from agents that remain investigational; each tier is summarized in a comparative table of results in all-comers versus results in older/unfit patients and limitations. Advances in reduced-intensity conditioning and GVHD prophylaxis have extended allogeneic transplantation to select fit older patients, while low-dose chemotherapy to manage cell count and supportive care remain vital for non-aggressive pathways. **Conclusions:** In our review, we have outlined current treatment options for older AML patients, with an emphasis on distinguishing which regimens are supported by dedicated evidence in this population, particularly those unfit for IC.

## 1. Introduction

Acute myeloid leukemia (AML) is the most prevalent subtype of leukemia disorders and occurs predominantly in the elderly population, with a median diagnostic age of 68 years [1]. Epidemiologic data demonstrate a marked increase in disease incidence with advancing age, escalating from approximately 1 case per 100,000 at 40 years to greater than 15 cases per 100,000 in individuals aged 70 years or older [2]. Although newer therapeutic approaches have emerged, survival outcomes remain limited, with a 5-year overall survival (OS) of roughly 30% and a further decline to 5–10% in patients diagnosed beyond 65 years of age [3].

Advanced age is an unfavorable prognostic factor commonly linked to high-risk biological and disease characteristics. Prognosis declines with each decade starting in the late 30s, and older AML patients face higher induction mortality and reduced chemotherapy tolerance due to comorbidities and limited reserve [4]. Older patients are at increased risk of presenting with secondary AML (sAML), which occurs from pre-existing myelodysplastic syndrome (MDS) or therapy-related AML (t-AML) [5]. The ELN 2022 guidelines risk-stratify AML based on genetic abnormalities at the time of diagnosis of AML and divide patients into favorable, intermediate, and adverse risk categories. This risk stratification can especially help guide the treatment plan for geriatric patients [6]. Approximately 30 to 33% of elderly AML patients are considered suitable for intensive treatment. However, the general prognosis for this population is still poor, and the long-term benefit of intensive chemotherapy (IC) is still marginal. Older adults experience substantially poorer outcomes than younger patients when treated with IC. Induction-related mortality ranges from 10 to 20%, complete remission is achieved in only 45 to 65% of patients, and among those who attain remission, long-term survival is approximately 15% [7].

Functional assessment using performance status (PS) scales is integral to therapeutic decision-making in oncology and is commonly performed using the Karnofsky performance status (KPS) or Eastern Cooperative Oncology Group (ECOG) criteria to evaluate chemotherapy tolerance. A KPS of 80% or an ECOG PS of less than 2 is a favorable PS [1]. The traditional 7 + 3 IC, which consists of cytarabine and an anthracycline, has shown subpar results in patients over 75. A score of ECOG PS ≥ 3 correlates with a higher chance of death with the use of IC. Elderly patients over the age of 60 and with an ECOG PS of 2 undergoing IC demonstrated a 31% risk of 30-day mortality [8]. Reflecting these risks, NCCN guidelines recommend avoiding IC in patients with an ECOG PS > 2 [9]. For older AML patients in whom IC is not advisable, the combination of hypomethylating drugs (HMAs) such as azacitidine and venetoclax, a BCL-2 inhibitor, represents a promising option for improving clinical outcomes [4].

Allogeneic hematopoietic stem cell transplantation (alloHSCT) remains the only potentially therapeutic management for patients with intermediate- and adverse-risk AML. Despite this, many older patients are unable to proceed to alloHSCT because of advanced age, frailty, and significant comorbidities [10,11]. The main objective of treatment in older individuals is to achieve complete remission (CR), maintain CR with consolidation or maintenance therapy, and maintain a good quality of life. The ELN 2022 guidelines provide standardized response criteria and outcome measure definitions for AML clinical trials, which help evaluate therapeutic efficacy and guide future research and clinical implementation [6].

This review will focus on current treatment options for AML in elderly patients and the development of novel therapies that can be used in the elderly who cannot tolerate conventional IC. To directly address the need for a clearer, comparative perspective on the evidence base, Section 3 below organizes agents into three explicit evidence tiers—those with dedicated results in older/unfit patients, those approved for AML but without devoted older patient-specific data, and those that are still investigational—each summarized in a Table 1, Table 2, Table 3, and Table 4 respectively.

## 2. Tools to Assess Fitness and Disease Burden in Elderly AML Patients Undergoing Chemotherapy

Functional status reflects an individual’s ability to independently perform routine daily tasks. The Activities of Daily Living (ADL) and Instrumental Activities of Daily Living (IADL) scales are well-validated tools commonly used to assess functional capacity. In a small cohort of older patients with AML, impairment in IADL emerged as the strongest predictor of OS [12]. Prior studies have also demonstrated that both age at diagnosis and PS are closely linked to early (30 days) treatment-related mortality in AML, with death increasing markedly with advancing age and declining functional ability, reaching 50% in patients older than 75 years with a PS of 2 and 82% in those with a PS of 3 [8].

The comprehensive geriatric assessment (CGA) is a multidimensional tool employed by physicians to assess comorbidities, cognitive function, social support, functional capacity, nutritional status, and medication use [13]. Oncologists can use the CGA results to predict outcomes and guide therapy for older patients. A study involving 101 people aged 65 and older with newly diagnosed AML studied whether geriatric assessment (GA) characteristics, such as vigorous exercise, pain, and baseline comorbidity score, served as independent mortality predictors, even with ECOG PS scores of 0 to 1, thereby substantiating the assertion that GA enhances the utility of standard PS assessment [14]. Despite this, there is no consensus on which GA tools to use, optimal timing, or how to apply the results in routine cancer management [1].

Marconi et al. evaluated the influence of baseline comorbidities on overall survival in a large cohort of 131 elderly AML patients who were uniformly allocated to therapy with hypomethylating drugs (HMAs) using a comorbidity score such as the augmented comorbidity index (A-CI) and AML-CM scores. Hypoalbuminemia (*p* < 0.001), a marker of inflammatory state and lung disease (*p* = 0.013), had a significant influence on the prognosis. Of paramount relevance, having a high comorbidity load (>6 concurrent comorbidities) appears to be the most significant predictor of OS [3]. Ferrara et al. advocated using new criteria of unfitness for both intensive and non-intensive treatment in AML patients. The authors suggested that unfitness for IC should be evaluated using criteria that consider other systemic disorders [15]. Comorbidity screening should be included in daily clinical practice and research studies as part of the inclusion and exclusion criteria.

The decision to treat older AML patients based on disease biology, baseline comorbidity, and host-related variables is becoming increasingly crucial. It may result in improved patient outcomes, a higher quality of life, reduced psychological and social strain, and lower healthcare-related expenses. Multiple tools and measures discussed above are regularly used in clinical practice to better understand patients’ baseline disease burden and fitness before starting chemotherapy.

## 3. Current Therapeutic Options for Elderly Patients with AML

A wide range of drugs have shown activity in AML, but not all of them have been studied specifically in older or unfit patients.

**Table 1:** Summary of genetic mutations, their altered pathogenesis, and novel therapeutics approved or under investigation in AML.

Target/Gene Mutation	Altered Pathogenesis	Associated Therapies
FLT3 gene (FLT3-ITD, FLT3-TKD)	FLT3 gene mutations cause constitutive activation of the tyrosine kinase receptor	First-generation TKI: midostaurin, sorafenib. Second-generation TKI: gilteritinib, quizartinib, crenolanib
IDH1 & IDH2 genes	Alteration in the Krebs cycle increases production of 2-HG, which blocks myeloid differentiation	IDH1: ivosidenib, olutasidenib. IDH2: enasidenib
CD47	Cell-membrane protein that sends a “don’t-eat-me” signal to macrophages	Magrolimab
CD123	Cell-surface protein overexpressed on leukemic stem cells	Antibody–drug conjugate: pivekimab; CAR-T; bispecific T-cell engagers: vibecotamab, flotetuzumab
Menin (KMT2A fusion, NPM1, NUP98)	Alteration of HOX gene expression driving leukemogenesis	Revumenib, ziftomenib, bleximenib, enzomenib, BN-104, BMF-219

Abbreviations: TKI—tyrosine kinase inhibitor; 2-HG—2-hydroxyglutarate.

### 3.1. Drugs Approved with Efficacy Data Specifically in Older or Unfit AML Patients

Intensive chemotherapy (IC) can provide clinical benefit in select patients older than 70 years who lack significant comorbid conditions [5]. However, historical studies with standard 7 + 3 IC showed a subpar median survival of <1 year at age ≥ 75 years and an increased likelihood of mortality within 30 days of treatment initiation [8]. Attempts to improve outcomes through anthracycline dose escalation have not been successful [16,17]. In the NCRI AML17 trial, Burnett et al. demonstrated similar complete remission (CR) rates (75% vs. 73%) and 2-year overall survival (OS) rates (60% vs. 59%) when comparing daunorubicin doses of 60 mg/m^2^ and 90 mg/m^2^ [16]. No survival advantage was observed with the higher dose in any age cohort, including patients aged 60–65 years, despite earlier reports by Löwenberg et al. indicating benefit in this subgroup [16,17].

In parallel, retrospective analyses of high-risk AML treated with high-dose cytarabine (HiDAC) in combination with mitoxantrone have shown modest efficacy, with combined CR and CR with incomplete hematologic recovery (CRi) rates near 55% [18]. IC may be explored as a curative-intent treatment for fit elderly patients with favorable-risk AML according to ELN 2022 guidelines [6]. However, careful assessment of performance status, cognitive functions, social circumstances, and comorbidities is required [12,13,14,15]. This approach remains particularly relevant in core-binding factor (CBF) AML, where durable remission without allogeneic hematopoietic stem cell transplantation (alloHSCT) has been observed [19]. Specifically, a retrospective study by Mosna et al. reported a 5-year OS of 44.2% (95% CI, 39.9–47.5) and a 5-year event-free survival (EFS) of 32.9% (95% CI, 25.5–40.1) in older CBF-AML patients receiving intensive treatment [19].

CPX-351 is a liposomal preparation that delivers cytarabine and daunorubicin in a synergistic 5:1 molar ratio and is approved for the treatment of secondary AML (sAML) based on a pivotal randomized phase III trial reported by Lancet et al. [20,21]. The formulation extends intracellular drug exposure time, and the liposome carrier enables the therapy to circumvent drug efflux pumps [20]. In the phase III trial, patients aged 60–75 years with newly diagnosed therapy-related AML (tAML), AML evolving from prior MDS or CML, or de novo AML with myelodysplasia-related cytogenetic abnormalities (AML-MRC) were randomized to receive CPX-351 or standard 7 + 3 IC [21]. CPX-351 demonstrated superior efficacy, leading to FDA approval, with improvements in median OS (9.56 vs. 5.95 months) compared with 7 + 3, as well as a higher likelihood of proceeding to alloHSCT (34% vs. 25%) [21]. Extended follow-up at five years confirmed the durability of this benefit, showing a median OS of 9.3 months in the CPX-351 group versus 5.95 months in the control arm, with corresponding 5-year OS rates of 18% and 8% [22]. CPX-351 is also used in younger patients with newly diagnosed tAML or AML-MRC; Lee et al. conducted a retrospective study demonstrating similar response rates and survival outcomes among both younger and older patients [23].

Cytosine analogs like azacitidine (AZA) and decitabine (DEC) are phosphorylated by deoxycytidine kinase within the cell and incorporated into DNA and RNA, resulting in DNA hypomethylation [24]. The clinical benefit of azacitidine in older AML patients was demonstrated in the AZA-AML-001 trial, which randomized patients to azacitidine administered for 7 days every 4 weeks versus conventional care regimens (CCRs), low-dose cytarabine (LDAC), or IC [25]. While rates of CR and CRi were similar between groups (27.8% with AZA vs. 25.1% with CCR, *p* = 0.5348), azacitidine significantly prolonged median OS (10.4 vs. 6.5 months, *p* = 0.0101) [25]. Similar results were observed in the DACO-016 trial, where decitabine given for 5 days every 4 weeks was compared with either LDAC or supportive care, yielding superior CR rates (17.8% vs. 7.8%, *p* = 0.001) and improved OS (7.7 months vs. 5.0 months, *p* = 0.037) [26]. These findings established hypomethylating agents (HMAs) as standard therapy for older AML patients unfit for conventional IC [25,26]. Importantly, HMAs demonstrate enhanced activity in high-risk disease, particularly TP53-mutated AML, where decitabine administered at 20 mg/m^2^ for 10 days per cycle produced overall response rates (ORRs) of 100% in TP53-mutated cases and 67% in patients with high-risk cytogenetic abnormalities [27].

Venetoclax selectively inhibits B-cell lymphoma-2 (BCL-2), an anti-apoptotic protein essential for leukemic cell survival [28]. Historically, options for older AML patients unfit for IC were limited to single-agent AZA or DEC, resulting in a median OS of only 7.7 to 10.4 months [25,26]. The incorporation of venetoclax into HMA backbones has fundamentally altered standard care [29]. In the landmark phase III VIALE-A trial, AZA combined with venetoclax was compared to AZA plus placebo in an unfit older cohort, yielding markedly higher CR/CRi rates (66.4% vs. 28.3%) and median OS (14.7 vs. 9.6 months) [29]. Notably, the trial protocol administered venetoclax for 28 days, which was associated with increased adverse events, highlighting the clinical necessity of individualizing and potentially shortening venetoclax duration based on patient tolerance [29]. Additionally, a phase 1b study evaluating venetoclax combined with AZA or DEC in older AML patients demonstrated a composite CR/CRi rate of 71%, with response rates reaching 82% in IDH1/2-mutated disease and 88% in adverse-risk cytogenetics [30]. A retrospective propensity score-matched trial comparing 10-day DEC plus venetoclax versus IC further supported VEN-based superiority, showing higher CR rates (81% vs. 52%), lower 30-day mortality (1% vs. 24%), and extended median OS (12.4 vs. 4.5 months) [31].

Gemtuzumab ozogamicin (GO), an anti-CD33 antibody–drug conjugate, is approved using fractionated low-dose schedules in non-intensive or attenuated settings [32]. In the ALFA-0701 trial, adding low-dose GO to therapy improved EFS and OS in CD33-positive older patients, particularly those with favorable or intermediate cytogenetics, though clinical use requires close monitoring for hepatic veno-occlusive disease (VOD/SOS) [32].

**Table 2:** FDA-approved drugs/regimens with efficacy data reported specifically in older or unfit AML patients.

Drug/Regimen (Trial)	Results in All-Comers (If Reported)	Results Specifically in Older/Unfit Patients	Limitations
Azacitidine monotherapy (AZA-AML-001) [25]	CR/CRi 27.8% vs. 25.1% with conventional care (*p* = 0.53)	Trial enrolled older, newly diagnosed AML patients (>30% blasts); mOS 10.4 vs. 6.5 months (*p* = 0.101)	CR benefit not statistically significant; superseded as backbone therapy by venetoclax-based combinations
Decitabine monotherapy (DACO-016) [26]	CR 17.8% vs. 7.8% vs. LDAC/supportive care (*p* = 0.001)	Population was older, newly diagnosed AML patients; OS 7.7 vs. 5.0 months (*p* = 0.037)	Modest OS benefit
Decitabine, 10-day extended dosing [27]	Not a randomized all-comer comparison	ORR 100% in TP53-mutated AML and 67% in high-risk cytogenetics	Small cohort; single-arm; benefit is response-based rather than survival-proven
Venetoclax + Azacitidine (VIALE-A) [29]	Not applicable—trial was enrolled specifically in an unfit/older cohort	Population ≥ 75 y or unfit for IC; mOS 14.7 vs. 9.6 months (HR 0.66, *p* < 0.001); CR/CRi 66.4% vs. 28.3%	28-day venetoclax dosing associated with increased adverse events; optimal duration still individualized
Venetoclax + Decitabine or Azacitidine, phase 1b [30]	Not applicable—enrolled ≥ 65 y ineligible for induction	CR/CRi 71% overall; 82% in IDH1/2-mutated disease; 88% in adverse-risk cytogenetics	Non-randomized, single-arm
Decitabine (10-day) + Venetoclax vs. IC, retrospective PSM [31]	Comparator arm was IC (CR 52%, 30-day mortality 24%, OS 4.5 months)	CR 81% vs. 52%; 30-day mortality 1% vs. 24%; OS 12.4 vs. 4.5 months	Retrospective, propensity-matched rather than randomized
CPX-351 [21,22,23]	Comparator was standard 7 + 3 (mOS 5.95 months)	Population 60–75 y with newly diagnosed t-AML/AML-MRC; mOS 9.56 vs. 5.95 months; 5-yr OS 18% vs. 8%; similar response/survival in younger vs. older patients	Benefit specific to secondary/MRC AML; early mortality remains substantial
Gemtuzumab ozogamicin, fractionated low-dose (ALFA-0701/0501) [32]	Not applicable—studied in older, lower-intensity settings	Improved EFS and OS in CD33+ older patients, particularly favorable/intermediate cytogenetics	Requires monitoring for hepatic veno-occlusive disease (VOD/SOS); less benefit in adverse cytogenetics
Ivosidenib + Azacitidine (AGILE) [33]	Comparator was placebo + azacitidine (mOS 7.9 months)	Population newly diagnosed IDH1-mutated, ≥75 y or IC-ineligible; mOS 24.0 vs. 7.9 months (HR 0.44); CR 47.2% vs. 14.9%	Benefit limited to the IDH1-mutated subset (~7–14% of AML)
Enasidenib monotherapy, phase 1/2 [34]	Not a randomized all-comer trial	Heavily pretreated R/R IDH2-mutated cohort, median age 68; ORR 40.3%, CR 19.3%	Modest CR rate; no randomized older-specific comparator
Intensive 7 + 3 chemotherapy, dose-finding trials [16,17,35]	CR 75% vs. 73%, 2-yr OS 60% vs. 59% for daunorubicin 90 vs. 60 mg/m^2^ (AML17)	No survival advantage to dose escalation in patients ≥ 60–65 y; median survival < 1 year at ≥75 y in prior series of 998 patients ≥ 65 y	Only ~30–33% of older patients are eligible; outcomes remain poor even when treated
IC in core-binding-factor (CBF) AML, retrospective [19]	Not applicable—CBF-specific series	Durable remission without alloHSCT achievable; 5-yr OS 44.2% (95% CI 39.9–47.5), 5-yr EFS 32.9% (95% CI 25.5–40.1)	Retrospective; applies to favorable-risk CBF subset only

### 3.2. Drugs Approved for AML but Lacking Dedicated Efficacy Data Specifically in Older or Unfit Patients

Mutations of the FLT3 gene, including internal tandem duplications (ITDs) and tyrosine kinase domain (TKD) point mutations, result in persistent activation of FLT3 signaling and contribute to leukemogenesis [36]. Under the 2022 ELN classification system, FLT3-ITD-positive AML is categorized as intermediate risk [6]. First-generation TKIs such as midostaurin and sorafenib inhibit a broad spectrum of kinases [37]. Second-generation FLT3 inhibitors—gilteritinib, quizartinib, and crenolanib—are significantly more selective and potent [38,39]. In the phase III RATIFY trial, adding midostaurin to 7 + 3 induction led to significant OS and EFS improvements in newly diagnosed FLT3-mutated AML; however, the trial enrolled a younger, fit cohort [40]. Quizartinib similarly gained approval alongside 7 + 3 induction in newly diagnosed FLT3-ITD AML following the QuANTUM-First trial, demonstrating extended median OS (31.9 vs. 15.2 months) in an IC-eligible population [39]. Because both midostaurin and quizartinib are approved exclusively in combination with intensive chemotherapy, their application in older or unfit patients remains strictly limited [39,40].

For relapsed or refractory (R/R) FLT3-mutated AML, gilteritinib monotherapy was approved based on the ADMIRAL trial, which established superior median OS (9.3 vs. 5.6 months) and higher CR/CRi rates (34% vs. 15%) compared to salvage chemotherapy [38]. While gilteritinib is well tolerated in older adults, ADMIRAL enrolled a broad adult population without specific age-stratified reporting in primary outputs [38].

IDH1 and IDH2 genes encode metabolic enzymes catalyzing the conversion of isocitrate to α-ketoglutarate [41]. Pathogenic mutations generate the oncometabolite 2-hydroxyglutarate (2-HG), disrupting epigenetic regulation, impairing myeloid differentiation, and suppressing cytochrome c oxidase activity to increase cellular BCL-2 dependence [41,42,43]. Selective inhibitors ivosidenib (IDH1) and enasidenib (IDH2) both received approval in R/R IDH-mutated AML [34,44]. Olutasidenib, another potent IDH1 inhibitor, earned approval for R/R IDH1-mutated AML based on durable complete remissions demonstrated by de Botton et al. [45]. However, registration trials for single-agent ivosidenib and olutasidenib in the R/R setting evaluated general cohorts without dedicated older-specific subgroup analysis [44,45].

Menin inhibitors represent a novel class of targeted agents designed to disrupt the interaction between menin and KMT2A [46]. Oncogenic KMT2A fusion proteins drive leukemogenesis through aberrant activation of homeobox (HOX) gene transcription, with menin serving as an essential cofactor [47,48]. NPM1-mutated AML is similarly characterized by dysregulated HOX expression [49,50,51]. In the Phase 1 AUGMENT-101 trial, revumenib achieved CR/CRh rates of 33% in KMT2A-rearranged and 21% in NPM1-mutated R/R leukemia [52]. Likewise, ziftomenib demonstrated a 30% CR rate in NPM1-mutated AML in the KO-MEN-001 trial [53,54]. Both agents received FDA approval in R/R KMT2Ar and NPM1-mutated AML [52,53]; nevertheless, primary registration data reflect broad R/R populations rather than fitness-stratified older groups [52,53].

**Table 3:** FDA-approved AML drugs whose pivotal evidence is not specific to older or unfit patients.

Drug (Trial)	Approval Basis/Trial Population	Reported Results	Why Older-Specific Evidence Is Limited
Midostaurin (RATIFY) [40]	Added to 7 + 3 induction in newly diagnosed FLT3-mutated AML; population largely younger, IC-fit adults	Improved OS (*p* = 0.009) and EFS (*p* = 0.002) vs. placebo + IC	Only approved in combination with intensive induction—not usable in patients who cannot receive IC
Gilteritinib monotherapy (ADMIRAL) [38]	R/R FLT3-mutated AML, general adult population, not restricted to older/unfit	mOS 9.3 vs. 5.6 months; CR/CRh 34% vs. 15%	Results not broken out for an elderly-specific subgroup in the primary report
Quizartinib + IC (QuANTUM-First) [39]	Newly diagnosed FLT3-ITD AML receiving intensive chemotherapy—by definition an IC-eligible (fitter) population	mOS 31.9 vs. 15.2 months	Not studied as monotherapy or specifically in unfit/older patients
Ivosidenib monotherapy, R/R [44]	R/R IDH1-mutated AML, general population	Durable remissions reported in early trials	Activity data come chiefly from a general R/R cohort, not an elderly-specific one
Olutasidenib, R/R [45]	R/R IDH1-mutated AML; population not reported by age	Durable complete remissions reported	No separate older-specific outcome data presented
Revumenib (AUGMENT-101) [52]	R/R KMT2A-rearranged and NPM1-mutated AML, general R/R population	CR/CRh 33% (KMT2Ar), 21% (NPM1m)	KMT2Ar and NPM1m are common in older adults, but the trial was not age-stratified
Ziftomenib (KOMET-001; early KO-MEN-001) [53,54]	R/R KMT2Ar/NPM1m AML, general population	CR 30% in NPM1-mutated AML	Same limitation—not restricted to or reported separately for older adults
High-dose cytarabine + mitoxantrone, retrospective [18]	“High-risk AML” cohort; age composition not clearly restricted to older adults	Combined CR/CRi~55%	Population age not clearly delineated; cannot confirm applicability to the elderly specifically

### 3.3. Investigational Agents and Combinations Under Study

Sorafenib was evaluated in combinations within the SORAML and ALLG AMLM16 trials, but results did not demonstrate sufficient clinical benefit to gain FDA approval [55,56]. To address the unfitness gap in FLT3-mutated AML, frontline gilteritinib plus azacitidine was evaluated in the Phase 3 LACEWING trial [57]. Although it yielded a significantly higher composite CR rate compared to azacitidine monotherapy (58.1% vs. 26.5%), median OS did not achieve statistical significance [57]. Triplet regimens combining FLT3 inhibitors with HMAs and venetoclax have shown high activity in early trials [58,59]. Phase 1/2 studies evaluating quizartinib + decitabine + venetoclax, as well as gilteritinib + azacitidine + venetoclax (reported by Short et al.), demonstrated high CR/CRi rates and favorable responses in unfit older adults [58,59].

In IDH-mutated disease, ivosidenib was approved with azacitidine in newly diagnosed unfit IDH1-mutated AML following the phase III AGILE trial, which showed marked OS improvement (24.0 vs. 7.9 months; HR 0.44), EFS prolongation (HR 0.33), and high mutation clearance [33]. Further extending this targeted approach, phase 1b/2 studies investigating triplet therapy with ivosidenib, venetoclax, and optional azacitidine yielded an ORR of 94%, a 3-year OS of 70.5% (95% CI, 57.7–86.1%), and a median EFS of 50.4 months [60,61].

CD47 is an immune-checkpoint cell membrane protein upregulated on AML blasts and leukemic stem cells (LSCs) that transmits a “don’t-eat-me” signal to macrophage SIRPα receptors [62,63]. Anti-CD47 monoclonal antibodies like magrolimab promote LSC phagocytosis [63]. A phase 1b trial combining magrolimab with azacitidine in unfit AML (median age 73 years; 65% TP53-mutated) showed promising activity, including a 71% ORR and 67% CR/CRi in the TP53-mutated subgroup, with median OS reaching 12.9 months in TP53-mutated and 18.9 months in TP53 wild-type patients [64]. However, subsequent phase 3 trials (ENHANCE-2 and ENHANCE-3) revealed significant safety signals involving hemolytic anemia, leading the FDA to place a clinical hold on the program [64].

CD123 (IL-3 receptor α chain) is overexpressed on LSCs relative to normal hematopoietic stem cells [65,66]. Pivekimab sunirine (PVEK) is an antibody–drug conjugate targeting CD123 [67]. Single-agent PVEK demonstrated modest activity (20% ORR) in R/R AML [67]. However, combination regimens have yielded strong efficacy: PVEK + AZA + VEN achieved a 51% ORR in R/R disease and impressive CR/CRc rates of 52%/66% in frontline unfit CD123+ AML, with 73% of complete responders attaining measurable residual disease (MRD) negativity [68,69]. Other CD123-targeted modalities under investigation include bispecific T-cell engagers (vibecotamab, flotetuzumab) and off-the-shelf CAR-T therapies (UCART123v1.2 in the Ameli-01 trial) [70,71]. Tagraxofusp, an IL-3-diphtheria toxin fusion protein approved for BPDCN, acts by inhibiting elongation factor-2 [72,73]. While tagraxofusp monotherapy shows modest AML activity, combining it with azacitidine and venetoclax has yielded promising clinical results [74].

**Table 4:** Investigational agents and combinations under study (not FDA-approved for AML).

Agent/Combination	Target/Mechanism	Available Results	Status
Sorafenib [55,56]	FLT3 (first-generation TKI)	SORAML and ALLG AMLM16 trials did not demonstrate sufficient benefit	Not approved
Gilteritinib + Azacitidine, frontline (LACEWING) [57]	FLT3i + HMA, in ND FLT3m AML unfit for IC	Composite CR 58.1% vs. 26.5% with azacitidine alone; OS difference not statistically significant	Investigational/off-label
Quizartinib + Decitabine + Venetoclax [58]	FLT3i + HMA + BCL-2i triplet	High CR/CRi rates reported in a phase 1/2 study	Phase 1/2
Gilteritinib + Azacitidine + Venetoclax [59]	FLT3i + HMA + BCL-2i triplet	Favorable outcomes reported in an updated phase 1/2 study	Phase 1/2
Ivosidenib + Venetoclax ± Azacitidine [60,61]	IDH1i + BCL-2i ± HMA triplet	ORR 94%; 3-yr OS 70.5% (95% CI 57.7–86.1); median EFS 50.4 months	Phase 1b/2
Magrolimab + Azacitidine [64]	Anti-CD47 antibody + HMA	In a TP53-mutated subgroup (median age 73), objective response 71%, CR/CRi~67%; mOS 12.9 months (TP53m) vs. 18.9 months (TP53 wild-type)	Program placed on FDA hold after a hemolytic anemia signal in the ENHANCE-2/3 trials
Pivekimab (±azacitidine/venetoclax) [67,68,69]	CD123-targeting antibody–drug conjugate	ORR 20% as monotherapy; ORR 51%/CRc 31% with AZA + VEN in R/R AML; CR/CRc 52%/66% frontline, 73% MRD-negative among responders	Phase 1/2
CD123 bispecific T-cell engagers (vibecotamab, flotetuzumab) [70]	CD123 & CD3 engagers	Have not demonstrated sufficient efficacy to date	Early phase/discontinued programs
CD123 CAR-T (UCART123v1.2, Ameli-01) [71]	CD123-directed allogeneic CAR-T	Early feasibility data only	Phase 1
Tagraxofusp ± azacitidine/venetoclax [72,73,74]	CD123-IL-3–diphtheria toxin fusion; approved for BPDCN only	Modest as monotherapy in AML; promising early results in combination with AZA/VEN in ND and R/R AML	Investigational in AML

Abbreviations: TKI—tyrosine kinase inhibitor; HMA—hypomethylating agent; BCL-2i—BCL-2 inhibitor; ORR—overall response rate; CRc—composite complete remission; MRD—measurable residual disease.

### 3.4. AlloHSCT in the Older Patient

Allogeneic hematopoietic stem cell transplantation should remain part of treatment planning for selected older adults with AML rather than being excluded based on chronological age alone [75,76]. Eligibility should be individualized according to physiologic fitness, comorbidity burden, organ function, disease risk, response status, donor availability, social support, and patient goals. Reduced-intensity conditioning and non-myeloablative conditioning have increased the feasibility of transplantation in older adults, although relapse and non-relapse mortality remain important for competing risks. Expansion of the alternative donor pool, including greater use of mismatched, unrelated, and haploidentical donors using post-transplant cyclophosphamide (PTCy) for GVHD prophylaxis, has further increased transplant access for appropriately selected older patients [75,76].

### 3.5. Supportive, Palliative-Intent, and Low-Intensity Symptom-Control Options

Not every older adult with AML is an appropriate candidate for intensive or prolonged disease-directed therapy. When antileukemic treatment is not appropriate or is inconsistent with the patient’s goals, best supportive management should be discussed explicitly within a shared decision-making framework [75]. Supportive care may include red-cell transfusion for clinically significant anemia, platelet transfusion according to bleeding risk and platelet count, and prevention or treatment of infection according to the clinical setting [6,75]. For patients who are no longer receiving antileukemic therapy, including those receiving end-of-life or hospice care, ASH suggests that red-cell transfusions remain available; platelet transfusions may be appropriate in selected circumstances such as clinically significant bleeding [75]. When hyperleukocytosis or leukostasis requires prompt cytoreduction, hydroxyurea is commonly used to lower the white blood cell count [6]. Low-intensity antileukemic therapy with LDAC (18% CR vs. 1% with hydroxyurea in the AML14 trial) would only be appropriate if the goals of care shifted toward some antileukemic benefit, given its added treatment burden [77].

## 4. Future Directions

### 4.1. Monitoring of Minimal Residual Disease (MRD) and Its Implementation in AML

MRD is a series of very sensitive laboratory procedures capable of identifying residual leukemic cells even below the morphologic level. It can currently detect as low as 10^−6^ AML blasts [78,79]. MRD assessment in AML is used to predict impending relapse, guide timely therapeutic interventions, provide a quantitative measure of deep remission, serve as a surrogate endpoint to accelerate drug development and approval, and facilitate post-remission risk stratification [80]. Currently, the two most widely reviewed techniques are molecular MRD (Mol-MRD) assessed using qPCR and multiparameter flow cytometry-based MRD (MFC-MRD) [81]. NGS and dPCR (digital PCR) are two other technologies [82]. Maiti et al. studied the role of MRD in low-intensity treatment in older unfit AML patients. Importantly, patients who attained MRD-negative status experienced markedly improved outcomes, including significantly longer median overall survival (25.1 vs. 7.1 months, *p* < 0.001) [83]. The prognostic value of MRD was further validated in the VIALE-A trial, which evaluated patients treated with venetoclax and azacitidine. In this cohort, approximately 41% of patients achieved MRD negativity at a threshold of <10^−3^, and multivariable analysis identified complete remission with MRD < 10^−3^ as a strong predictor of OS (*p* < 0.001) [84]. Ongoing investigations continue to explore the role of MRD assessment in refining risk stratification and guiding management decisions in elderly AML populations.

### 4.2. TP53

The *TP53* gene encodes p53, a phosphoprotein that functions as a transcription factor central to cell cycle regulation, DNA damage repair, and cell death [85]. *TP53* plays an important role in regulating self-renewal and development of normal hematopoietic stem cells; its mutation causes excessive pluripotent stem cell production, leading to leukemogenesis [86]. *TP53*-mutated AML has a poor prognosis and a limited response to conventional therapy, with alloHSCT achieving durable remission in just 10–15% of patients [87]. Cytotoxic IC in eligible patients with *TP53*-mutated AML has shown a brief OS of 5–9 months and a dismal response of 28–42% [87]. Welch et al. demonstrated improved clinical responses and substantial, though incomplete, clearance of *TP53* mutations in patients with AML and MDS characterized by adverse-risk cytogenetics and *TP53* alterations [27]. A subsequent meta-analysis by Daver et al. evaluating treatment-naïve *TP53*-mutated AML reported higher CR rates with IC and with venetoclax plus HMAs compared with HMA monotherapy (43% vs. 33% vs. 13%); however, these improvements did not translate into a significant OS advantage across treatment groups [88]. Similarly, DiNardo et al. reported a CR/CRi rate of 47% in *TP53*-mutated AML treated with VEN combined with decitabine or azacitidine, with a median OS of 7.2 months and a median duration of response of 5.6 months, reflecting only incremental gains over historically reported response rates as low as 18% [89]. The traditional IC, HMAs, and venetoclax therapy for *TP53*-mutated AML have demonstrated short-lived and dismal outcomes. Therefore, trials including immune-related therapies such as immune checkpoint inhibitors, anti-CD47 antibodies, bispecific and dual-antigen receptor antibodies, CD123- and CD70-targeted agents, and CAR T-cell therapy alone or in combination with HMAs/VEN are ongoing.

### 4.3. Current Trials Ongoing for the Treatment of AML in the Elderly

Table 5 depicts a summary of selected ongoing trials of novel therapeutics, HMAs, and VEN. Results of these trials are highly anticipated, as they will guide future treatment plans for AML in the elderly.

**Table 5:** Selected ongoing clinical trials in AML relevant to older/unfit patients.

ClinicalTrials.gov ID	Phase	Treatment	Study Population	Endpoint (Primary)	Ref.
NCT04913922	II	Relatlimab + Nivolumab + 5-Azacitidine	R/R AML, ND AML in patients unfit for IC	MTD, DLTs, ORR	[90]
NCT06317649	II	Gilteritinib + AZA + VEN vs. AZA + VEN alone	Older and Unfit Adults With ND FLT3-m AML	Rate of MRD-negative CR	[91]
NCT04140487	I/II	Gilteritinib + AZA + VEN	ND and R/R AML/HR MDS	MTD of gilteritinib and ORR	[92]
NCT03690115	II	Ponatinib	FLT3-ITD AML in cytologic CR (post Allo-SCT)	Relapse incidence at 2 years from transplant	[93]
NCT04603001	I	LY3410738 (IDH1/2 inhibitor) + AZA + VEN	IDH-m R/R AML, MDS, and other hematologic malignancies	MTD of LY3410738 and ORR	[94]
NCT03471260	Ib/II	IVO + VEN ± AZA	IDH-m MDS, MD AML, R/R AML	ORR, DLT, and incidence of AEs	[95]
NCT02677922	Ib/II	Enasidenib + AZA vs. AZA alone, IVO + AZA vs. AZA alone	IDH-1/2m ND AML	ORR, DLT, and incidence of AEs	[96]
NCT03113643	I	Tagraxofusp + AZA + VEN	CD-123 R/R AML, treatment-naïve AML	MTD	[97]
NCT04065399	I/II	Revumenib	MLL/KMT2A-r or NPM1-m R/R AML	DLTs, TEAEs	[98]
NCT04067336	I/II	Ziftomenib	KMT2A-r or NPM1-m R/R AML	MTD, incidence of AEs, minimal biologically effective dose, evidence of anti-leukemia activity	[99]
NCT06384261	II	AZA + VEN vs. AZA + VEN + Cusatuzumab	ND AML in patient unfit for IC	Overall Survival	[100]

MRD—measured residual disease; MTD—maximum tolerated dose; CR—complete remission; ORR—objective response rate; DLT—dose-limiting toxicity; TEAE—treatment-emergent adverse event; AE—adverse event.

## 5. Conclusions

The management of AML in older patients continues to present difficult challenges that must be addressed. Proper guidelines for selecting appropriate treatments are crucial to avoid adverse effects of high-intensity medications like traditional 7 + 3 IC. Notably, introducing HMAs and VEN has proven beneficial in treating elderly patients unfit for IC. PARADIGM, a pivotal randomized phase II study by Fathi et al. recently published in the New England Journal of Medicine (originally presented at the plenary session at the American Society of Hematology meeting in December 2025), compared 7 + 3 or CPX-351 to AZA + VEN for all patients and highlighted the improved response rates and higher number of patients undergoing alloHSCT in the AZA + VEN arm [101]. Knowing that most patients in this study had adverse-risk disease characteristics, the results of this study make us rethink the definition of older or elderly patients with AML. With 75 years as the cutoff defined in VIALE-A, in the real world many patients over the age of 60 with adverse risk characteristics are now getting HMA + VEN-based regimens; this is the era of individualizing treatment regimens and prioritizing clinical trials rather than a one-size-fits-all approach.

Novel therapeutics targeting specific genetic mutations and monoclonal antibodies with reduced toxicity as single agents or in triplet combinations with HMA + VEN are currently being developed, with encouraging initial results for mutations such as FLT3 and IDH1/2. However, challenges remain in developing effective treatments for adverse-risk cytogenetics like TP53. Allogeneic HSCT remains the only potentially curative approach in this population, and its expanding use even among older, higher-risk patients underscores the need for ongoing, individualized fitness assessment rather than age-based exclusion. Our review highlights frontline therapy for older patients with AML with minimal coverage of AML with adverse-risk cytogenetics or relapsed/refractory AML, consolidation treatment after induction chemotherapy, and nuances of AlloHSCT. Future studies are needed to provide evidence-based strategies to improve prognosis for older adults with AML unfit for IC. For patients in whom antileukemic therapy is no longer appropriate or consistent with their goals, a well-defined transition to supportive care, including transfusion support and prompt cytoreduction when needed, should be incorporated into shared decision-making as an equally essential part of AML management. Ultimately, optimal and individualized management of AML in the elderly depends on assessing patient fitness and selecting a suitable regimen that maximizes quality of life, improves social and mental well-being, and enhances the likelihood of durable remission.

## Data Availability

No new data were created or analyzed in this study. Data sharing is not applicable to this article.
